# Progress in Neurology

**Published:** 1898-07-02

**Authors:** 


### Progress in Neurology.

**Anatomy.**—Bruce[1] gives an interesting description of two tracts of fibres in the posterior columns of the lumbo-sacral region of the spinal cord, containing endogenous fibres, which are so named because they are derived from cells in the cord itself, and do not originate in the posterior roots. One of these tracts, named the cornu-commisural tract, lies in the anterior part of the posterior column in close apposition to the posterior commisure and septum. The second, the septo-marginal tract, has a close relation to the posterior median septum. The first-mentioned tract does not degenerate in locomotor ataxy even in its advanced stage, nor in cases of injury or compression of the cauda equina, whilst it does degenerate in conditions which lead to atrophy or degeneration of the cells of the posterior cornu. The tract attains its greatest size at the level of the lower lumbar region, diminishing somewhat rapidly above, and more gradually below this level. The septo-marginal tract is of triangular shape, occupying the postero-internal angle of the posterior column, and extends from the lowest sacral root to the twelfth dorsal root, and attains its maximum size at the junction of the sacral with the lumbar region of the cord. As regards the cells from which these two tracts originate, it is not possible as yet to make a definite statement; nor can anything be asserted with certainty as to their function except that they are probably commisural. Their position suggests a connexion with the lower organic reflexes.

Risien Russel[2] has made an experimental research on the innervation of the muscles concerned in the production of spasmodic wry-neck, which has important bearings on the operative measures which should be undertaken to remedy that distressing condition. Unless only the muscles innervated by the spinal accessory are affected, it is perfectly useless to divide that nerve. Division of the posterior branches of the upper cervical nerves is the only remedy which offers a reasonable prospect of permanent relief in severe cases. It is hence important to know the precise position in which the head is placed by contraction of the group of muscles supplied by each individual nerve root. Excitation of the first and second cervical nerves produces an almost pure lateral inclination of the head on the shoulder; the second produces, in addition, a very slight backward movement of the head, and the slightest possible rotation on the vertical axis. Stimulation of the third and fourth cervical nerves produces a movement of the head which is chiefly backward, the occiput being drawn to the excited side, and the chin, in consequence, turned away from the stimulated side and upwards. Excitation of the fifth and sixth roots causes remarkably little movement of the head; what there is consists in backward retraction of the occiput. The seventh and eighth cervical roots produce, under electrical stimulation, much less movement of the head than the four upper cervical roots, but much more than the fifth and sixth. The effect is to pull the occiput backwards and to the side stimulated, but the movement is secondary, being effected by contraction of the latissimus dorsi, which pulls the scapula and muscles connected with it downwards. There is thus a striking pairing of the nerve roots as regards movement of the head—the first and second produce the same movement, the third and fourth another movement, the fifth and sixth are almost inactive as regards head movement, and the seventh and eighth each produce an indirect effect. It follows that surgical treatment for wry-neck must be chiefly directed to the four upper nerve roots, and, according to the predominant type of movement, more especially to the first and second or to the third and fourth. On the question whether after section of the main supply of a muscle, a smaller subsidiary supply may in time conduct all impulses, the author admits that whilst, as a rule, definite parts of a muscle are supplied by separate nerve roots, yet after section of the nerve roots the muscle may recover mobility to a remarkable extent, even when there is no possibility of reunion taking place between the divided ends of the root. Risien Russel[3] has also made experimental investigations into the origin and destination of some of the afferent and efferent tracts of the medulla oblongata. He finds that the descending antero-lateral tract in the spinal cord which degenerates after lesion of the lateral region of the medulla, has its source of origin in Deiter's nucleus, as first described by Ferrier and Turner. Fibres derived from the restiform body proper, and degenerating caudalwards after section of that structure, do not form an efferent tract in the spinal cord. The tract described by Marchi as degenerating in the spinal cord after lesions of the cerebellum is due to accidental lesion of Dieter's nucleus. There is no evidence that the "direct sensory cerebellar tract" of Edinger is composed of afferent fibres, but it appears to be an efferent tract from the nucleus globosus to the nucleus of Dieter.

**Colour-hearing.**—Many years ago Galton showed that a considerable percentage of persons have the faculty of experiencing a sensation of colour in association with certain sounds, the colour seen being definite and invariable for the same sound. Colman[4] finds that the cases fall into two groups. In the first there is a crude colour sensation associated with certain sounds, such as each of the vowel sounds or musical notes. The appearance is usually that of a transparent coloured film in front of the observer, not obscuring objects. In the second group there are colour sensations whenever letters or written words were spoken or thought of, so that when a word is uttered the subject visualises the letters, each having a distinctive tint. These phenomena are associated sensations, analogous, for instance, with shivering at the sound of the squeak of a slate pencil. Their origin is very obscure; they nearly always date back to early childhood. It has been suggested that they are due to learning the alphabet from a coloured picture book, but this is certainly not so in all cases. The colours do not vary as time goes on. For instance, in one case they were exactly the same after ten years. Comparison of the associated colours as seen by various individuals shows that they are hardly ever the same, *i.e.*, that the same sound is associated with a different colour in the case of each person. The phenomenon cannot, therefore, depend on any physical relationship between sound and colour; the process is a psychical one.

There is a close association between these colour sensations and the symbols which many people associate with abstract ideas, and with the mental diagrams which always occur with others in connection with numbers, dates, and serial events.

These phenomena are most prominent in early life, and tend to fade if disregarded. They may be helpful for simple processes of spelling, learning by rote, mental arithmetic; but for higher mental operations, *e.g.*, higher mathematics, abstract speculation, they are likely to prove a hindrance, and so should not be encouraged in children.

**Migraine.**—John Mitchell[5] has published a case of migraine in which elaborate unusual phenomena occurred. The patient first sees a tiny dwarf appearing at a great distance. This figure gradually approaches, becoming larger and larger, finally assuming the form of a gigantic figure with a club. During his approach the headache gradually increases, and when he comes close he seems to strike the patient repeatedly on the head with the club, producing excruciating pain until consciousness is lost. Violent convulsions usually follow, with arching of the body so extreme as to cause it to rest on the head and heels. The attack may last as long as eight hours, from the first appearance of the dwarf to loss of consciousness, but is usually less.

**Meniere's Disease.**—De la Tourette[6] reports the case of a man, aged 58 years, who was taken suddenly one morning with a violent vertigo having all the characters of Menière's disease. He was given quinine in large doses with excellent results on the vertigo. This treatment, which was first advocated by Charcot, seems to be little known, though it is by far the best means at our disposal for treating the vertigo of this complaint. The dose of quinine is 10 grains once or twice a day for a period of at least a fortnight.

**Locomotor Ataxy.**—Obersteiner[7] remarks that the exact origin of the disease is still doubtful. He inclines to the view that it should be classed as a tertiary manifestation of syphilis, though probably exposure to cold, traumatism, and poisons can call forth or help to bring about the appearance of the disease, even in the absence of syphilis. In the morbid anatomy the questions arise as to whether the whole of the direct prolongation of the posterior roots in the cord degenerates. The chief fact which has been established is that whereas the intramedullary portion of the posterior root fibres degenerate, that portion which is outside the cord—*i.e.*, between the posterior root ganglion and the cord—shows very little change. At the place where the roots enter the cord the medullary sheath is reduced to a minimum, and it is possibly a place of less resistance. Here a meningeal lesion or altered blood vessels would suffice to cause degeneration. A primary interstitial overgrowth can no longer be accepted as a sufficient explanation.

Williamson[8] has had an opportunity of examining the spinal cord in an early case of locomotor ataxy, three years after the onset only, where death had been due to an accident. Well-marked sclerosis in the posterior columns of the cord was found in all regions, though most marked in the lumbar region. The other columns of the cord were not affected. In the lumbar region the sclerosis was most marked between the caput of the posterior horn and the entrance of the posterior intermediate septal artery into the white matter of the cord. The median portion of Goll's columns was less affected, especially at the posterior two-thirds. The anterior part of the posterior columns was almost normal. (*Cp.* Bruce's investigations, described above.) The walls of the artery were much thickened in the sclerosed region. The nerve-roots between the posterior root ganglia and the cord were but little affected; there appeared to be a slight increase of the interstitial connective tissue, but no other change—no degenerating nerve fibres—could be seen. The pia mater of the posterior portion of the cord in the lumbar region presented a slight increase in the number of round cells, and was a little increased in thickness. The pathological changes found in this case do not support the views of Leyden, Marie, and others, that the changes in the cord are secondary to disease in the nerve roots due to an altered condition of the cells of the posterior root ganglia.

[1] Bruce, Brain, 1897. [2] Risien Russel, Brain, 1897. [3] Risien Russel, Proc. Roy. Soc., 1897. [4] Colman, Lancet, Jan. 1, 1898. [5] Mitchell, Amer. Jour. Nerv. Dis., Nov. 1897. [6] De la Tourette, Sem. Medic., No. 33, 1897. [7] Obersteiner, Berlin Klin. Wochen, Oct. 18, 1897. [8] Williamson, Med. Chron., Oct. 1897.

